# Surprising trunk rotational capabilities in chimpanzees and implications for bipedal walking proficiency in early hominins

**DOI:** 10.1038/ncomms9416

**Published:** 2015-10-06

**Authors:** Nathan E. Thompson, Brigitte Demes, Matthew C. O'Neill, Nicholas B. Holowka, Susan G. Larson

**Affiliations:** 1 Department of Anatomical Sciences, Stony Brook University, Health Sciences Center T8-040, Stony Brook, New York 11794-8081, USA; 2 Department of Basic Medical Sciences, University of Arizona College of Medicine, Phoenix, Health Sciences Education Building, Phoenix, Arizona 85004-2157, USA; 3 Interdepartmental Doctoral Program in Anthropological Sciences, Stony Brook University, Social & Behavioral Sciences Building, Stony Brook, New York 11794-4364, USA

## Abstract

Human walking entails coordinated out-of-phase axial rotations of the thorax and pelvis. A long-held assumption is that this ability relies on adaptations for trunk flexibility present in humans, but not in chimpanzees, other great apes, or australopithecines. Here we use three-dimensional kinematic analyses to show that, contrary to current thinking, chimpanzees walking bipedally rotate their lumbar and thoracic regions in a manner similar to humans. This occurs despite differences in the magnitude of trunk motion, and despite morphological differences in truncal ‘rigidity' between species. These results suggest that, like humans and chimpanzees, early hominins walked with upper body rotations that countered pelvic rotation. We demonstrate that even if early hominins walked with pelvic rotations 50% larger than humans, they may have accrued the energetic and mechanical benefits of out-of-phase thoracic rotations. This would have allowed early hominins to reduce work and locomotor cost, improving walking efficiency early in hominin evolution.

## Abstract (web summary)

Greater trunk flexibility in humans is thought to be a major adaptation to bipedal walking compared to chimpanzees. Here Thompson *et al*. show that chimpanzees are capable of human-like trunk rotations during bipedalism, suggesting bipedal proficiency was present in early hominins.

## Graphical abstract

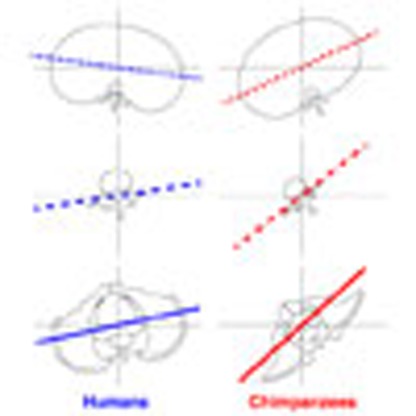

Human walking involves coordinated motion of the pelvis, lumbar region and thorax. Pelvic rotations allow for longer steps^1^,^2^,^3^,^4^, while the out-of-phase rotation of the thorax reduces angular momentum of the trunk^5^,^6^,^7^. This intra-trunk motion additionally facilitates arm swing out of phase with the legs, further reducing angular momentum^7^,^8^,^9^. By utilizing intrinsic segmental cancellations of angular momentum during bipedal locomotion, humans are able to reduce muscular work^7^ and locomotor cost^10^,^11^,^12^. It has long been assumed that the ability to utilize rotatory motions depends on a derived human-like tall trunk and narrow waist^13^,^14^,^15^,^16^.

Our closest living relatives, chimpanzees, exhibit markedly divergent trunk morphology when compared with modern humans. Tall and wide iliac blades, a short lumbar vertebral column, and a large, laterally flaring lower rib cage^17^ all contribute to a trunk configuration that has often been portrayed as rigid and immobile^14^,^18^,^19^,^20^,^21^. This morphological rigidity implies restricted motion between the thorax and pelvis in all anatomical planes, and would thus prohibit out-of-phase axial rotations of the thorax and pelvis during bipedal locomotion^14^,^15^,^19^.

Reconstructions of the earliest well-represented hominins have indicated similarities in trunk morphology to chimpanzees^22^,^23^. In particular, *Australopithecus afarensis* (as exemplified by ‘Lucy', A.L. 288-1) possesses a wide pelvis^24^ and a wide, inferiorly flaring lower rib cage^22^,^25^. Consequently, it has been hypothesized that *Au. afarensis* and perhaps other early hominins may have also lacked the ability of the thorax to counter axial pelvic rotations during bipedal locomotion^15^,^19^,^25^. This would suggest that only with the advent of more modern trunk morphology, which likely emerged in *Homo erectus*^15^,^16^,^26^,^27^,^28^,^29^, would these counter rotations have been able to occur. However, this evolutionary scenario is premised on the notion that the chimpanzee trunk is rigid, a hypothesis which, to date, has not been tested *in vivo* in bipedally walking chimpanzees.

Here we used three-dimensional (3D) kinematics to empirically test the hypothesis that the trunk of chimpanzees is rigid in the transverse plane (axial rotations) during bipedal locomotion. We divided the trunk into three anatomical segments (thorax, lumbar region and pelvis) and measured the overall range of motion (ROM) of these segments in the transverse plane relative to a global coordinate system (absolute ROM), and the ROM of these segments relative to one another (relative ROM). The term ‘out of phase' is used to refer to two segments that rotate in opposite directions at the same time (that is, one segment has a positive angle and one has a negative angle in a global coordinate system), while the term ‘in phase' is used to refer to two segments that rotate in the same direction at the same time.

Our results show that chimpanzees walking bipedally display larger absolute ROM's of all trunk segments compared with humans, and an in-phase pattern of motion between the pelvis and thorax, compared to the out-of-phase pattern in humans. Despite this, relative to the pelvis, thoracic and lumbar motion was remarkably similar between humans and chimpanzees. These results show that chimpanzees do not have rigid trunks, as has been hypothesized on the basis of osteology alone. Further, differences in trunk morphology compared with humans do not prevent the thorax from countering pelvic motion, and the magnitude of pelvic motion dictates whether thoracic rotations are in- or out-of-phase with the pelvis. We find that human-like out-of-phase pelvic and thoracic rotations were likely possible in early hominins even if their pelves rotated 50% more than those of modern humans.

## Results

### Range of motion comparisons

**Figure 1:**
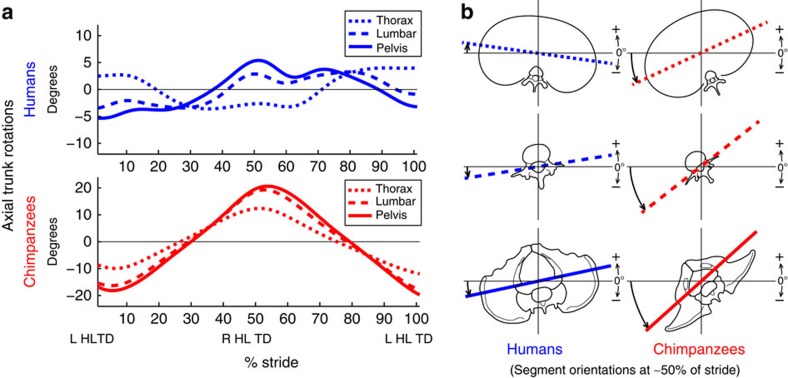
Mean angular motion of all segments for humans and chimpanzees over a stride. (**a**)Rotations are relative to a global coordinate system. Note the difference in *y* axis scale between species. LHLTD and RHLTD represent left and right hind limb touchdowns, respectively. (**b**) Angular motions near 50% of stride for humans and chimpanzees with segment motion represented by transverse lines (rotations exaggerated to enhance clarity). The chimpanzee thorax remains in phase with the pelvis, in contrast to the out-of-phase relationship in humans.

Overall absolute ROM was significantly larger in chimpanzees than in humans for all trunk segments (Wilcoxon rank-sum test, *P*<0.001, *n*=50 and 14 strides for humans and chimpanzees, respectively, for all interspecific comparisons, Table 1, Fig. 1; individual subject data in Supplementary Figs 1 and 2). Within humans, females exhibited a greater ROM of all trunk segments compared with males (Wilcoxon rank-sum test, *P*<0.01 for the lumbar region and thorax, *n*=25 strides for both males and females), but these differences were slight in comparison with the differences between species (Supplementary Table 1). In both humans and chimpanzees, the lumbar region rotates in phase with the pelvis. The thorax in chimpanzees also rotates in phase with the pelvis, in contrast to the out-of-phase rotation in humans. However, although the thorax rotates in the same direction as the pelvis in chimpanzees, it rotates much less than does the pelvis (26.9±10.0° versus 42.8±10.5°).

**Figure 2:**
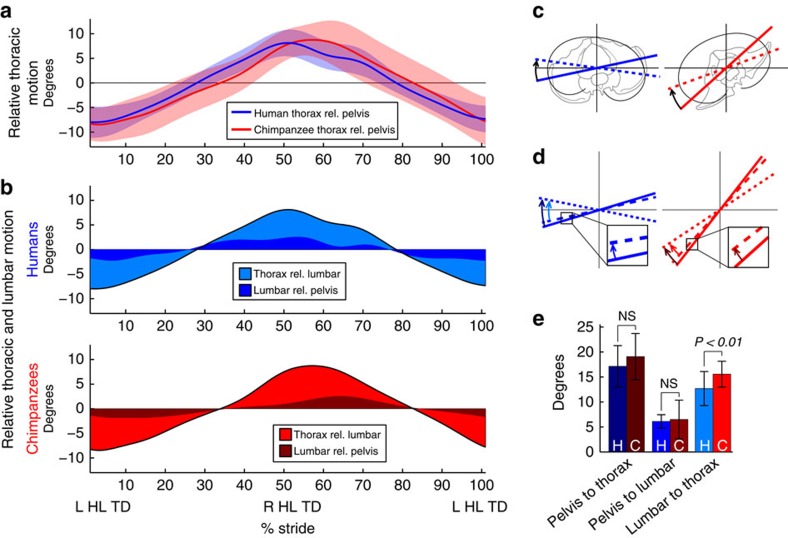
Relative motion of segments to one another in humans and chimpanzees. (**a**) Motion of the thorax relative to the pelvis over a stride (mean±s.d.). (**b**) Relative pelvis-to-thorax motion partitioned by the contributions of the lumbar and thoracic segments. (**c,d**) Angular motions near 50% of stride for humans and chimpanzees with segment motion represented by transverse lines (rotations exaggerated to enhance clarity). (**e**) Total range of relative pelvis-to-thorax, pelvis-to-lumbar and lumbar-to-thorax motion over a stride (mean±s.d.). H and C represent humans and chimpanzees, respectively; NS represents non-significance using a Wilcoxon rank-sum test at the *P*=0.05 level.

Despite these differences in absolute ROM, the relative motion between the thorax (inclusive of lumbar region motion) and the pelvis was nearly identical in pattern and comparable in magnitude in humans and chimpanzees (Fig. 2a; relative ROM: 17.1±4.1° and 19.1±4.6°, respectively; Wilcoxon rank-sum test, *P*=0.15). In both species, relative to the pelvis, the thorax was maximally rotated in opposition to the pelvis just after hind limb touchdown (that is, an absolute pelvic rotation to the right produced a relative thoracic rotation to the left). Although the magnitude of the relative pelvis-to-thorax motion was slightly larger in chimpanzees, the human mean fell within one s.d. of the chimpanzee mean for the entire stride.

Figure 2b,e further partitions relative pelvis-to-thorax motion into the constituent pelvis-to-lumbar, and lumbar-to-thorax components. Surprisingly, the contribution of these components is remarkably similar in humans and chimpanzees (Table 1; Supplementary Tables 1 and 2). In both species, pelvis-to-lumbar and lumbar-to-thorax motion comprises ∼28–33% and 67–72% of total pelvis-to-thorax motion, respectively. Between humans and chimpanzees, the relative motion between the pelvis and the lumbar region is nearly identical (6.1±1.3° and 6.5±3.9°, respectively). Although neither the range of pelvis-to-thorax nor pelvis-to-lumbar motion is significantly different between humans and chimpanzees, lumbar-to-thorax motion is significantly larger in chimpanzees (relative ROM: 12.7±3.4° for humans; 15.6±2.6° for chimpanzees; Wilcoxon rank-sum test, *P*<0.01). This difference is largely accounted for by slight differences between species in timing of maximum pelvis-to-lumbar motion, compared with overall pelvis-to-thorax motion (compare dark blue and dark red curves of Fig. 2b).

The motion between trunk segments that occurs in chimpanzees demonstrates that the chimpanzee trunk is not a rigid, immobile unit during bipedal walking. Chimpanzees achieve a reduction in upper body motion by rotating the thorax and lumbar region less than the pelvis. However, the absolute rotation of the chimpanzee pelvis is so pronounced that the reduction in pelvis-to-thorax motion still yields thoracic rotation in phase with the pelvis, in contrast to the out-of-phase rotation in humans (Fig. 2c,d).

### In-phase or out-of-phase relative motion

**Figure 3:**
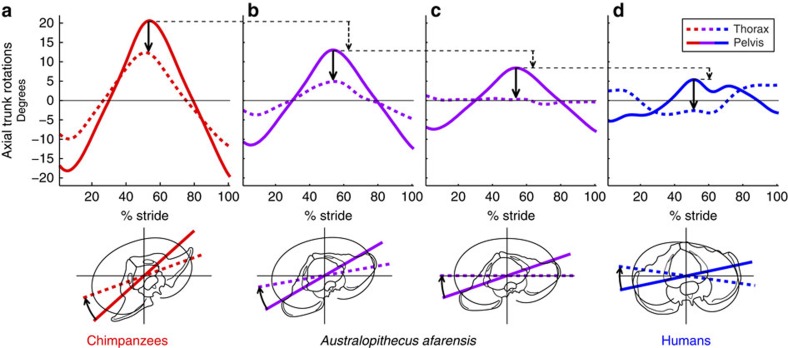
Hypothetical transitional forms of pelvic and thoracic motions in australopithecines compared with chimpanzees and humans. (**a**,**d**) Angular pelvic and thoracic motion for humans and chimpanzees (as in Fig. 1). Solid black arrows represent relative motion between thoracic and pelvic segments near midstride. Assuming a constant value for the offset between the two segments (average of human and chimpanzee curves in Fig. 2a), pelvic and thoracic motions were modelled by iteratively decreasing chimpanzee pelvic rotations and calculating thoracic rotations by subtracting the offset. A continuum of solutions is achieved with two shown here. (**b**) The point at which the magnitude of pelvic rotations is halfway between humans and chimpanzees (∼138% larger than the human curve in **d**). At this point, thoracic rotations remain in phase with the pelvis. (**c**) The point at which thoracic rotations switch from in phase to out of phase with the pelvis, as determined by the lowest root mean squared value of thoracic rotations in all iterations. Pelvic rotations are ∼53% larger than those of humans at this point. Axial pelvic rotations smaller than this result in an out-of-phase pelvis-to-thorax relationship (**d**).

The similarity in relative thoracic motion between humans and chimpanzees suggests that the absolute amount of pelvic motion may ultimately dictate whether the thorax moves in phase or out of phase with the pelvis. On the basis of this premise, we took the magnitude of pelvic rotations as a starting point, and calculated the magnitude and phasing of the resulting thoracic rotations. We modelled this relationship by starting with a chimpanzee-like magnitude of pelvic motion, and iteratively decreasing it to a human-like level. At each iteration, the average of the relative thoracic rotation angle found in both chimpanzees and humans (Fig. 2a) was subtracted from the pelvic angle (represented by the solid arrow of Fig. 3). Thoracic rotations decreased with each iteration until a threshold magnitude of pelvic motion was reached, at which point the thorax switched from an in-phase to an out-of-phase relationship with the pelvis (Fig. 3c). We calculated the pelvic ROM at this point as 16.5° (∼50% larger than human pelvic rotations; Fig. 3d). Pelvic rotations larger than this result in an in-phase relationship with the thorax, while pelvic rotations smaller than this result in an out-of-phase relationship.

## Discussion

Our results show that overall, chimpanzees exhibit larger absolute rotations of all trunk segments during bipedal walking in comparison with humans. However, the motion of the thorax and lumbar region relative to the pelvis show that despite marked differences in trunk morphology between humans and chimpanzees, chimpanzees are still able to use as much intra-trunk motion as humans. Especially surprising is the similarity in lumbar motion relative to the pelvis, where axial rotations are only 0.4° different between humans and chimpanzees. Thus, in terms of axial rotations, the chimpanzee lumbar region is not as rigid as has been portrayed on the basis of osteological features alone^18^,^20^,^21^. Despite fewer^30^ and shorter^31^,^32^ lumbar vertebrae—the last one or two of which are entrapped between tall iliac blades—the chimpanzee lumbar vertebral column still makes an important rotatory contribution during bipedal locomotion.

Although motion of the thorax relative to the pelvis is similar between species, motion of the thorax relative to the lumbar region is significantly larger in chimpanzees compared with humans (2.9°). These data suggest that the flaring lower thoracic morphology characteristic of chimpanzees and other great apes does not engender rotational rigidity of the trunk. Nor do they support the notion that the great ape-like dorsal placement of the iliac blades and abdominal oblique musculature would have prevented torsional rotations within the trunk during bipedalism^25^. The chimpanzee thorax is quite capable of attenuating rotations of the pelvis, even though its rotation remains in phase with that of the pelvis.

These results show that chimpanzees utilize thoracic and lumbar rotations to counter pelvic rotations in much the same way as humans. The main difference between species is the total ROM of each segment, and the phasic relationship between the pelvis and thorax. Humans utilize small ranges of pelvic motion and can achieve out-of-phase motion between the pelvis and thorax, whereas chimpanzees walk bipedally with large pelvic rotations and exhibit in-phase motion (Fig. 1). The in-phase relationship between the pelvis, lumbar region, and thorax in chimpanzees means that chimpanzees cannot utilize thoracic motion to counteract angular momentum of the pelvis to the degree that humans can, but the attenuation of motion seen in chimpanzees likely does generate some reduction in angular momentum as compared with walking with a fully rigid trunk. It may be the case that the chimpanzee thorax is countering pelvic motion to the greatest degree possible given their musculoskeletal anatomy. In other words, chimpanzees walking bipedally may be utilizing their full, or nearly full, ROM at each intervertebral level, whereas humans do not^33^,^34^.

Our data represent the first detailed 3D kinematic data of trunk motion during bipedalism in an ape. A previous study seeking to characterize trunk rotations in bipedal gibbons (*Hylobates pileatus*) found little-to-no relative motion between the thorax and pelvis^14^. The gibbon trunk shows some similarities with great apes, but differs in having a longer waist, typically possessing five lumbar vertebrae as in humans^30^, thus causing us to expect some counter rotatory ability. However, that study was of a zoo animal that was not outfitted with kinematic markers and, given the inherent difficulties of capturing 3D segment motion^35^, the authors may not have fully captured the actual transverse plane rotations. Alternatively, it is possible that gibbons utilize different trunk mechanics than either humans or chimpanzees. In either case, these findings reinforce the need to test hypothesized form-function relationships with empirical experimental data.

On the basis of similarities in trunk morphology with chimpanzees, *Au. afarensis* has been hypothesized as lacking an ability to rotate the thorax to counter pelvic rotations^15^,^19^,^25^. However, the striking similarity in relative pelvis-to-thorax motion in humans and chimpanzees suggests that some ability of the thorax to counter pelvic rotations was also present in early hominins. As chimpanzees are capable of within-trunk motion, differences in thoracic and pelvic shape compared with humans would likely not have precluded this ability in *Au. afarensis*. It should be noted that a second, larger individual assigned to *Au. afarensis* (KSD-VP-1/1)^36^, as well as the partial *Ardipithecus ramidus* skeleton (ARA-VP-6/500)^37^ have both been reconstructed as having a more human-like thoracic shape. Both reconstructions are ultimately based on partial upper ribs (*Au. afarensis*: second rib; *Ar. ramidus*: first rib), which may be of limited use in reconstructing the lower thoracic shape in hominins^23^. Cross-sectional dimensions of several unassociated mid-rib fragments from Hadar have added to criticism of the chimpanzee-like thoracic reconstruction of *Au. afarensis*^38^, and a consensus on thoracic shape in early hominins awaits further investigation. Nevertheless, if some early hominins are found to have a more human-like thoracic cage morphology than has been reconstructed for Lucy (A.L. 288-1)^22^, this would support our conclusion that intra-trunk motion was possible in early hominins during bipedal walking.

Our data also suggest that whether or not the australopithecine thorax moved fully out of phase with the pelvis as in humans may have largely depended on the magnitude of their pelvic rotations during walking. Interpretations of pelvic motion during bipedal locomotion in *Au. afarensis* vary greatly. On the basis of hypothesized trunk and hip function, some have proposed greater pelvic motion in *Au. afarensis* compared with humans^24^,^39^,^40^,^41^. In addition, a 3D forward dynamic modelling study of Lucy's gait also predicted larger axial pelvic rotations than in humans^42^, but how much larger is still uncertain. Forward dynamic solutions suggest that transverse plane hip rotation was 2.5 times larger in Lucy than in modern humans (50° versus 20°, respectively)^42^. The extent to which this might be due to independent rotation of the femur versus rotation of the pelvis is unclear, although it is likely largely driven by the latter. The representation of the upper body was also necessarily lumped into the ‘pelvic' segment in that model^42^. If pelvic motion in australopithecines was as much as 2.5 times that of modern humans, it would have likely resulted in an in-phase rotation of the pelvis and thorax (Fig. 3b). Conversely, others have proposed near or fully human-like pelvic kinematics for australopithecines^20^,^43^,^44^,^45^,^46^, which would suggest out-of-phase motion between the pelvis and thorax (falling somewhere along the spectrum from Fig. 3c,d). If so, australopithecines would have been able to utilize thoracic counter rotations to reduce whole-body angular momentum^7^,^9^, and to drive a passive, human-like arm swing during locomotion.

Although it is difficult to judge the total range of intervertebral motion available to early hominins, if having a great ape-like thoracic shape entailed intervertebral rotations that were near maximum during bipedal walking (as may be the case in chimpanzees), this may have been a limiting factor for behaviours that place a higher demand on within-trunk rotations. Running, for instance, which likely involves a larger degree of motion between the pelvis and thorax^15^,^47^, and larger cancellations of angular momentum^48^, may have been somewhat less effective.

The continuum of pelvis–thorax coupling presented here on the basis of chimpanzees and humans is not meant to imply that the last common ancestor of these species exhibited chimpanzee-like bipedal kinematics. However, it is notable that trunk motion in Japanese macaques also shows similarities to our measured values of chimpanzees. Three-dimensional kinematic data (although with a less detailed marker set than used here) indicate ranges of pelvis-to-thorax and hip rotation in macaques^49^ that are similar in magnitude to the chimpanzee values presented here and elsewhere^50^. Although their model lacks direct thoracic markers, Ogihara *et al*.^49^ also find an in-phase relationship between the pelvis and thorax, although perhaps with less absolute thoracic rotation. This suggests that the in-phase pattern of pelvic and thoracic motion in chimpanzees is not unique among nonhuman primates, and that the out-of-phase pattern in humans is likely the result of a derived reduction in pelvic rotation during bipedalism within hominins. At present, the question as to why humans walk bipedally with smaller pelvic rotations compared with other primates remains unanswered. The larger pelvic motions in nonhuman primates may be due to musculoskeletal differences such as iliac blade orientation and gluteal muscle position^50^, and/or may be necessary to increase stride length^42^,^49^,^50^ while walking with short, flexed lower limbs^50^.

A more exact understanding of the relationship between thoracic and pelvic motion and the existence of angular momentum cancellations in fossil hominins may await future experimental and modelling studies of bipedal walking. However, our data show that regardless of the precise reconstruction of thoracic shape and lumbar length in the last common ancestor and other fossil hominins, counter rotations of the thorax and pelvis would have been feasible early in the evolution of human bipedalism.

## Methods

### Experimental subjects and protocol

All experimental procedures in this study were approved by the Stony Brook Institutional Animal Care and Use Committee and the Stony Brook University Institutional Review Board. The 3D kinematic data of the trunk were collected in five male and five female humans and two male chimpanzees (*Pan troglodytes*) during bipedal walking (ages and body masses in Table 1). Each human subject provided written informed consent before participating in the experiment. Subjects walked bipedally across an 11-m runway. Humans were instructed to walk at a comfortable (that is preferred) speed, and were given ample practice time before trials were recorded. Chimpanzees walked bipedally either of their own accord, or following a trainer carrying a juice reward. Chimpanzees had been trained to walk bipedally for ∼2 years before recording sessions and were reasonably proficient at this behaviour; therefore, our results should reflect their inherent capability at bipedal walking. Five trials were recorded for each human subject and seven trials for each chimpanzee subject.

### Kinematic data collection and analysis

Marker clusters were used to define three trunk segments: thorax (thoracic vertebrae and ribs), lumbar region (lumbar vertebrae) and pelvis (Supplementary Table 3). The thorax and lumbar region were each defined by three markers. The pelvis was defined by at least three markers, with redundant markers used to increase accuracy. Some pelvic markers were not visible for large portions of a stride for chimpanzees. In these cases, those markers were removed from pelvic motion calculation for that stride. However, in all cases, at least three markers were visible for the entire stride, and in all but one case at least four markers were used to calculate pelvis motion. For the chimpanzees, markers were painted on the skin using non-toxic paint. For humans, lightweight ball markers were used. Marker positions were determined by palpation of anatomical landmarks.

Marker positions were recorded digitally using a four-camera motion capture system (Xcitex Inc., Woburn, MA, USA) at 150 frames per second. Marker positions were digitized in the software ProAnalyst (Xcitex, Inc.), and the resulting *x*, *y* and *z* coordinates were filtered with a fourth order, 6-Hz Butterworth filter as determined appropriate on the basis of a visual inspection of the filtered versus unfiltered data. For some trials (19 of 64), a single marker point was seen by only one camera for a few frames (typically between 1–7 frames). In these cases small gaps in tracking in other cameras were filled via point interpolation in ProAnalyst. When present, these gaps typically occurred with pelvic markers, where redundant markers (>3) ensured accurate kinematics. Transverse plane rotations in a global coordinate system were calculated over the stride using Cardan angles with the KineMat toolbox (http://isbweb.org/software/movanal/kinemat/) as well as custom-written code in MATLAB (The Mathworks Inc., Natick, MA, USA). Cardan angles were calculated using the sequence of rotations for spinal motion recommended by the International Society of Biomechanics^51^. A static image of each subject standing bipedally was used to determine the neutral position of each segment (0°). To correct for slight differences in standing posture between subjects and experimental days for plotting purposes, mean segment motion (Figs 1, 2, 3) was centred about 0° of motion, though some slight asymmetry has been noted in chimpanzee pelvic rotations previously^50^. However, s.d.'s were calculated from the original, un-centred data (see Supplementary Figs 1 and 2 for un-centred data). The kinematic methodology used here produced values similar to those of other human studies on pelvic, lumbar and thoracic kinematics both from skin mounted markers^1^,^2^,^3^,^4^,^5^,^6^,^9^, and bone pin markers^34^.

Linear speed for each trial was calculated using markers placed on the ischial tuberosity (chimpanzees) and/or anterior superior iliac spine (humans and chimpanzees). To facilitate comparisons between species at different speeds, dimensionless velocity (*v* (*gl*)^−1/2^)^52^ was calculated for all trials, where *g* is gravitational acceleration and *l* is lower limb length (Table 1). Lower limb length was taken as the distance between the greater trochanter and the ground at midstance (when the greater trochanter was positioned vertically above the ankle). Although no attempt was made to match dimensionless speeds between humans and chimpanzees, the averages of both species were nearly identical (Table 1; Supplementary Tables 1 and 2). Equality in dimensionless velocities^52^ minimizes differences between species that are due to speed alone, an effect that has been demonstrated in human pelvic motion^2^,^3^,^4^,^9^,^47^,^50^. Differences between species were tested for significance using Wilcoxon rank-sum tests because several variables were not normally distributed.

### Modelling of pelvic and thoracic rotations

Modelling of pelvic and thoracic rotations (Fig. 3) was performed using MATLAB. Mean pelvic rotation over a stride for chimpanzees was taken as a starting point. The magnitude of pelvic rotation was iteratively reduced in increments of 0.1% of the initial magnitude. At each iteration, thoracic motion was calculated from pelvic motion using the average of the relative thoracic motion curves for humans and chimpanzees (average of the two curves in Fig. 2a), which remained constant for all iterations. The root mean squared (r.m.s.) value over the stride for thoracic motion was then calculated at each iteration. The r.m.s. value is a measure of dispersion of the data around a zero point, and therefore the iteration with the lowest thoracic r.m.s. value was taken as the point where thoracic rotations were at a minimum over a stride. This was also the point at which the thorax transitions from being largely in phase to largely out of phase with the pelvis.

## Additional information

**How to cite this article:** Thompson, N. E. *et al*. Surprising trunk rotational capabilities in chimpanzees and implications for bipedal walking proficiency in early hominins. *Nat. Commun.* 6:8416 doi: 10.1038/ncomms9416 (2015).

## Supplementary Material

Video Walking with chimps What can we learn from chimps swinging their hips? In this Nature Video, we investigate the walking style of our primate cousins, and see what they can teach us about our ambling ancestors.

Supplementary Information Supplementary Figures 1-2 and Supplementary Tables 1-3

## Figures and Tables

**Table 1:** Kinematic variables for both species.

	Humans		Chimpanzees		
	Mean	s.d.	Mean	s.d.	
Mass (kg)	61.5	7.7	34.9	1.0	
Age (years)	25.8	2.6	7.1	0.1	
Lower limb length (m)	0.88	0.04	0.43	0.01	
Speed (m s^−1^)	1.36	0.08	0.97	0.09	
Dimensionless velocity	0.46	0.02	0.47	0.04	
Number of strides	50		14		
					
**Axial range of motion (°)**					***P*** **value**
Pelvis	12.7	3.5	42.8	10.5	<0.001
Lumbar	10.1	2.8	39.5	9.7	<0.001
Thorax	9.7	3.0	26.9	10.0	<0.001
Thorax relative to pelvis	17.1	4.1	19.1	4.6	0.15
Lumbar relative to pelvis	6.1	1.3	6.5	3.9	0.68
Thorax relative to lumbar	12.7	3.4	15.6	2.6	<0.01

*P* values are results of Wilcoxon rank-sum tests with a sample size of 50 and 14 for humans and chimpanzees, respectively.

## References

[b1] Murray M. P., Drought A. B. & Kory R. C. Walking patterns of normal men. J. Bone Joint Surg. Am. 46, 335–360 (1964). 14129683

[b2] Nottrodt J. W., Charteris J. & Wall J. C. The effects of speed on pelvic oscillations in the horizontal plane during level walking. J. Hum. Mov. Stud. 8, 27–40 (1982).

[b3] Wagenaar R. C. & Beek W. J. Hemiplegic gait: a kinematic analysis using walking speed as a basis. J. Biomech. 25, 1007–1015 (1992). 1517261 10.1016/0021-9290(92)90036-z

[b4] van Emmerik R. E. A. & Wagenaar R. C. Effects of walking velocity on relative phase dynamics in the trunk in human walking. J. Biomech. 29, 1175–1184 (1996). 8872274 10.1016/0021-9290(95)00128-x

[b5] Stokes V. P., Andersson C. & Forssberg H. Rotational and translational movement features of the pelvis and thorax during adult human locomotion. J. Biomech. 22, 43–50 (1989). 2914971 10.1016/0021-9290(89)90183-8

[b6] Crosbie J., Vachalathiti R. & Smith R. Patterns of spinal motion during walking. Gait Posture 5, 6–12 (1997).

[b7] Herr H. & Popovic M. Angular momentum in human walking. J. Exp. Biol. 211, 467–481 (2008). 18245623 10.1242/jeb.008573

[b8] Elftman H. The function of the arms in walking. Hum. Biol. 11, 529–535 (1939).

[b9] Bruijn S. M., Meijer O. G., van Dieën J. H., Kingma I. & Lamoth C. J. C. Coordination of leg swing, thorax rotations, and pelvis rotations during gait: the organisation of total body angular momentum. Gait Posture 27, 455–462 (2008). 17669652 10.1016/j.gaitpost.2007.05.017

[b10] Ortega J. D., Fehlman L. A. & Farley C. T. Effects of aging and arm swing on the metabolic cost of stability in human walking. J. Biomech. 41, 3303–3308 (2008). 18814873 10.1016/j.jbiomech.2008.06.039 PMC2741013

[b11] Umberger B. R. Effects of suppressing arm swing on kinematics, kinetics, and energetics of human walking. J. Biomech. 41, 2575–2580 (2008). 18621376 10.1016/j.jbiomech.2008.05.024

[b12] Collins S. H., Adamczyk P. G. & Kuo A. D. Dynamic arm swinging in human walking. Proc. Biol. Sci. 276, 3679–3688 (2009). 19640879 10.1098/rspb.2009.0664 PMC2817299

[b13] Keith A. Hunterian lectures on man's posture: its evolution and disorders. Lecture II: the evolution of the orthograde spine. Br. Med. J. 1, 499–502 (1923). 20771062 10.1136/bmj.1.3247.499 PMC2316342

[b14] Schmid P. & Piaget A. Three-dimensional kinematics of bipedal locomotion. Z. Morphol. Anthropol. 80, 79–87 (1994).

[b15] Bramble D. M. & Lieberman D. E. Endurance running and the evolution of *Homo*. Nature 432, 345–352 (2004). 15549097 10.1038/nature03052

[b16] Wood B. & Collard M. The human genus. Science 284, 65–71 (1999). 10102822 10.1126/science.284.5411.65

[b17] Schultz A. H. in The Chimpanzee ed Bourne G. H. Vol. 1, 50–153Karger (1969).

[b18] Ward C. V. Torso morphology and locomotion in *Proconsul nyanzae*. Am. J. Phys. Anthropol. 92, 291–328 (1993). 8291620 10.1002/ajpa.1330920306

[b19] Schmid P. in From Biped to Strider eds Meldrum D. J., Hilton C. E. 49–62Kluwer Academic/Plenum Publishers (2004).

[b20] Lovejoy C. O. The natural history of human gait and posture: part 1, spine and pelvis. Gait Posture 21, 95–112 (2005). 15536039 10.1016/j.gaitpost.2004.01.001

[b21] Lovejoy C. O. & McCollum M. A. Spinopelvic pathways to bipedality: why no hominids ever relied on a bent-hip-bent-knee gait. Philos. Trans. R. Soc. Lond. B Biol. 365, 3289–3299 (2010). 20855303 10.1098/rstb.2010.0112 PMC2981964

[b22] Schmid P. Eine rekonstruktion des skelettes von A.L. 288-1 (Hadar) und deren konsequenzen. Folia Primatol. 40, 283–306 (1983). 6414917 10.1159/000156111

[b23] Schmid P. . Mosaic morphology in the thorax of *Australopithecus sediba*. Science 340, 1234598 (2013). 23580537 10.1126/science.1234598

[b24] Rak Y. Lucy's pelvic anatomy: its role in bipedal gait. J. Hum. Evol. 20, 283–290 (1991).

[b25] Schmid P. in Origine(s) de la Bipédie chez les Hominidés eds Coppens Y., Senut B. 225–234CNRS (1991).

[b26] Jellema L. M., Latimer B. & Walker A. in The Nariokotome Homo erectus Skeleton eds Walker A., Leakey R. 294–325Harvard University Press (1993).

[b27] Latimer B. & Ward C. V. in The Nariokotome Homo erectus Skeleton eds Walker A., Leakey R. 266–293Harvard Univ. Press (1993).

[b28] Ruff C. B. & Walker A. in The Nariokotome Homo erectus Skeleton eds Walker A., Leakey R. 234–265Harvard Univ. Press (1993).

[b29] Walker A. & Ruff C. B. in The Nariokotome Homo erectus Skeleton eds Walker A., Leakey R. 221–233Harvard Univ. Press (1993).

[b30] Williams S. A. Variation in anthropoid vertebral formulae: implications for homology and homoplasy in hominoid evolution. J. Exp. Zool. B Mol. Dev. Evol. 318, 134–147 (2011). 22532475 10.1002/jezb.21451

[b31] Jungers W. L. in The Lesser Apes: Evolutionary and Behavioral Biology eds Preuschoft H., Chivers D. J., Brockelman W. Y., Creel N. 146–169Edinburgh Univ. Press (1984).

[b32] Susanna I., Alba D. M., Almécija S. & Moyà-Solà S. The vertebral remains of the late Miocene great ape *Hispanopithecus laietanus* from Can Llobateres 2 (Vallè-Penedès Basin, NE Iberian Peninsula). J. Hum. Evol. 73, 15–34 (2014). 24953667 10.1016/j.jhevol.2014.05.009

[b33] Gregersen G. G. & Lucas D. B. An *in vivo* study of the axial rotation of the human thoracolumbar spine. J. Bone Joint Surg. Am. 49, 247–262 (1967). 6018729

[b34] Rozumalski A. . The *in vivo* three-dimensional motion of the human lumbar spine during gait. Gait Posture 28, 378–384 (2008). 18585041 10.1016/j.gaitpost.2008.05.005

[b35] Cappozzo A. . Position and orientation in space of bones during movement: experimental artefacts. Clin. Biomech. 11, 90–100 (1996). 10.1016/0268-0033(95)00046-1 11415604

[b36] Haile-Selassie Y. . An early *Australopithecus afarensis* postcranium from Woranso-Mille, Ethiopia. Proc. Natl Acad. Sci. USA 107, 12121–12126 (2010). 20566837 10.1073/pnas.1004527107 PMC2901440

[b37] Lovejoy C. O., Suwa G., Spurlock L., Asfaw B. & White T. D. The pelvis and femur of *Ardipithecus ramidus*: the emergence of upright walking. Science 326, 71e1–71e6 (2009). 19810197

[b38] Ward C. V., Kimbel W. H., Harmon E. H. & Johanson D. C. New postcranial fossils of *Australopithecus afarensis* from Hadar, Ethiopia (1990–2007). J. Hum. Evol. 63, 1–51 (2012). 22652491 10.1016/j.jhevol.2011.11.012

[b39] Berge C. in Origine(s) de la Bipédie chez les Hominidés eds Coppens Y., Senut B. 113–119CNRS (1991).

[b40] Berge C. How did the australopithecines walk? A biomechanical study of the hip and thigh of *Australopithecus afarensis*. J. Hum. Evol. 26, 259–273 (1994).

[b41] Ruff C. in Primate Locomotion: Recent Advances eds Strasser E., Fleagle J., Rosenberger A., McHenry H. 449–469Plenum Press (1998).

[b42] Nagano A., Umberger B. R., Marzke M. W. & Gerritsen K. G. M. Neuromusculoskeletal computer modeling and simulation of upright, straight-legged, bipedal locomotion of *Australopithecus afarensis* (A.L. 288-1). Am. J. Phys. Anthropol. 126, 2–13 (2005). 15386246 10.1002/ajpa.10408

[b43] Lovejoy C. O., Heiple K. G. & Burstein A. H. The gait of *Australopithecus*. Am. J. Phys. Anthropol. 38, 757–779 (1973). 4735528 10.1002/ajpa.1330380315

[b44] Lovejoy C. O. The origin of man. Science 211, 341–350 (1981). 17748254 10.1126/science.211.4480.341

[b45] Crompton R. H., Yu L., Weijie W., Günther M. & Savage R. The mechanical effectiveness of erect and "bent-hip, bent-knee" bipedal walking in *Australopithecus afarensis*. J. Hum. Evol. 35, 55–74 (1998). 9680467 10.1006/jhev.1998.0222

[b46] Crompton R. H. . Human-like external function of the foot, and fully upright gait, confirmed in the 3.66 million year old Laetoli hominin footprints by topographic statistics, experimental footprint-formation and computer simulation. J. R. Soc. Interface 9, 707–719 (2012). 21775326 10.1098/rsif.2011.0258 PMC3284127

[b47] Seay J. F., van Emmerik R. E. A. & Hamill J. Influence of low back pain status on pelvis-trunk coordination during walking and running. Spine 36, E1070–E1079 (2011). 21304421 10.1097/BRS.0b013e3182015f7c

[b48] Hinrichs R. N. Upper extremity function in running. II: angular momentum considerations. Int. J. Sport Biomech. 3, 242–263 (1987).

[b49] Ogihara N., Makishima H. & Nakatsukasa M. Three-dimensional musculoskeletal kinematics during bipedal locomotion in the Japanese macaque, reconstructed based on an anatomical model-matching method. J. Hum. Evol. 58, 252–261 (2010). 20060569 10.1016/j.jhevol.2009.11.009

[b50] O'Neill M. C. . Three-dimensional kinematics of the pelvis and hind limbs in chimpanzee (*Pan troglodytes*) and human bipedal walking. J. Hum. Evol. 86, 32–42 (2015). 26194031 10.1016/j.jhevol.2015.05.012

[b51] Wu G. . ISB recommendation on definitions of joint coordinate system of various joints for the reporting of human joint motion—part I: ankle, hip, and spine. J. Biomech. 35, 543–548 (2002). 11934426 10.1016/s0021-9290(01)00222-6

[b52] Alexander R. M. & Jayes A. S. A dynamic similarity hypothesis for the gaits of quadrupedal mammals. J. Zool. 201, 135–152 (1983).

