# Supplementary material for: Surprising trunk rotational capabilities in chimpanzees and implications for bipedal walking proficiency in early hominins
Source: Nat Commun. 2015 Oct 6;6:8416. doi: 10.1038/ncomms9416 (PMC4600717; doi:10.1038/ncomms9416)
Supplement: Supplementary Information — Supplementary Figures 1-2 and Supplementary Tables 1-3 [file ncomms9416-s1.pdf]

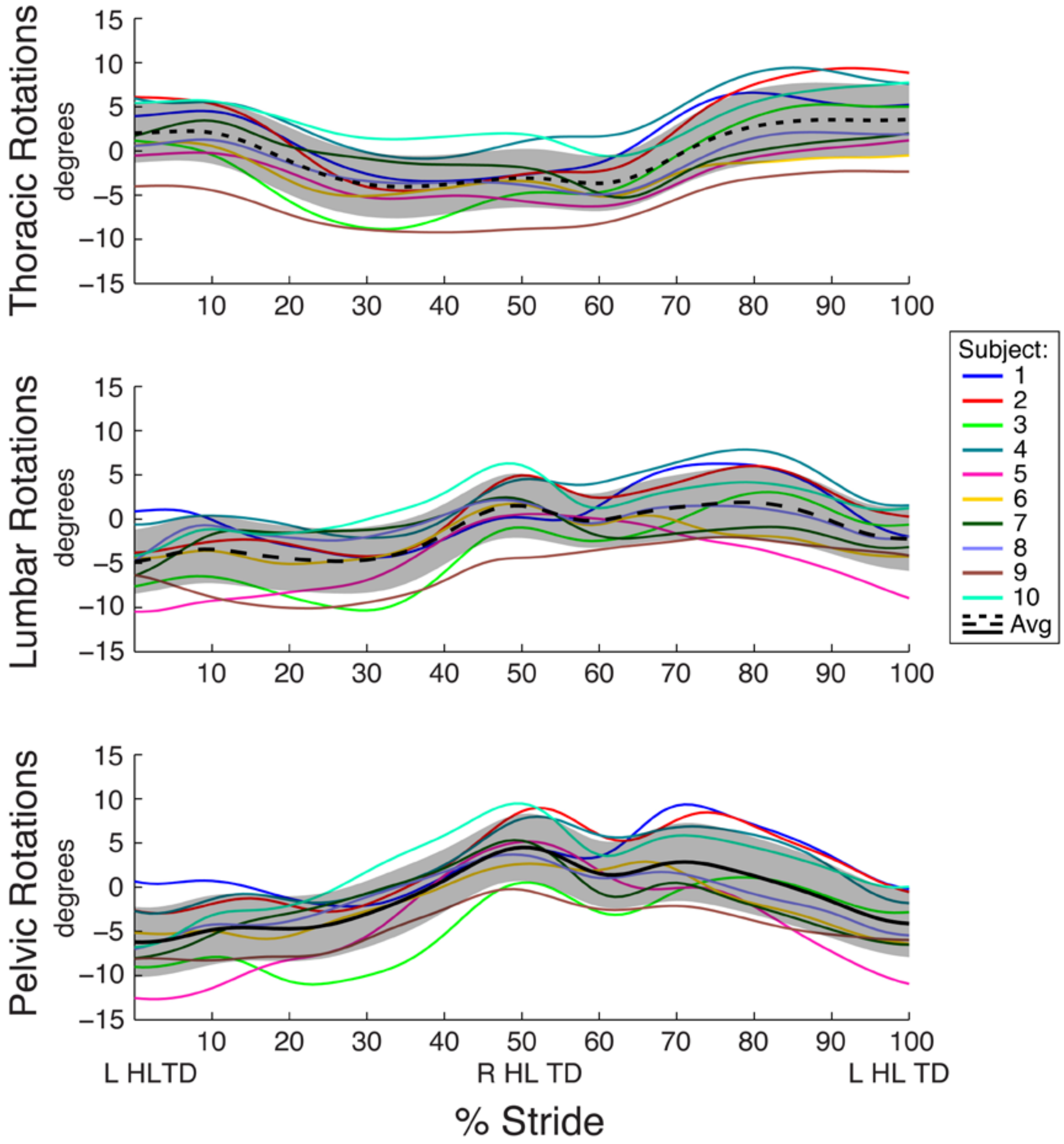

Supplementary Figure 1 | Mean angular motion of all segments for individual human subjects. Rotations are relative to a global coordinate system. Colored lines represent individual subject means, and the thick black line represents the mean for all human strides. Shaded area represents ± one standard deviation. Note that much of the variation between subjects relates not to shape of the curve, but to differences in y-axis offsets. This is largely due to slight variations in standing posture when taking the single image that defined the neutral position (0°).

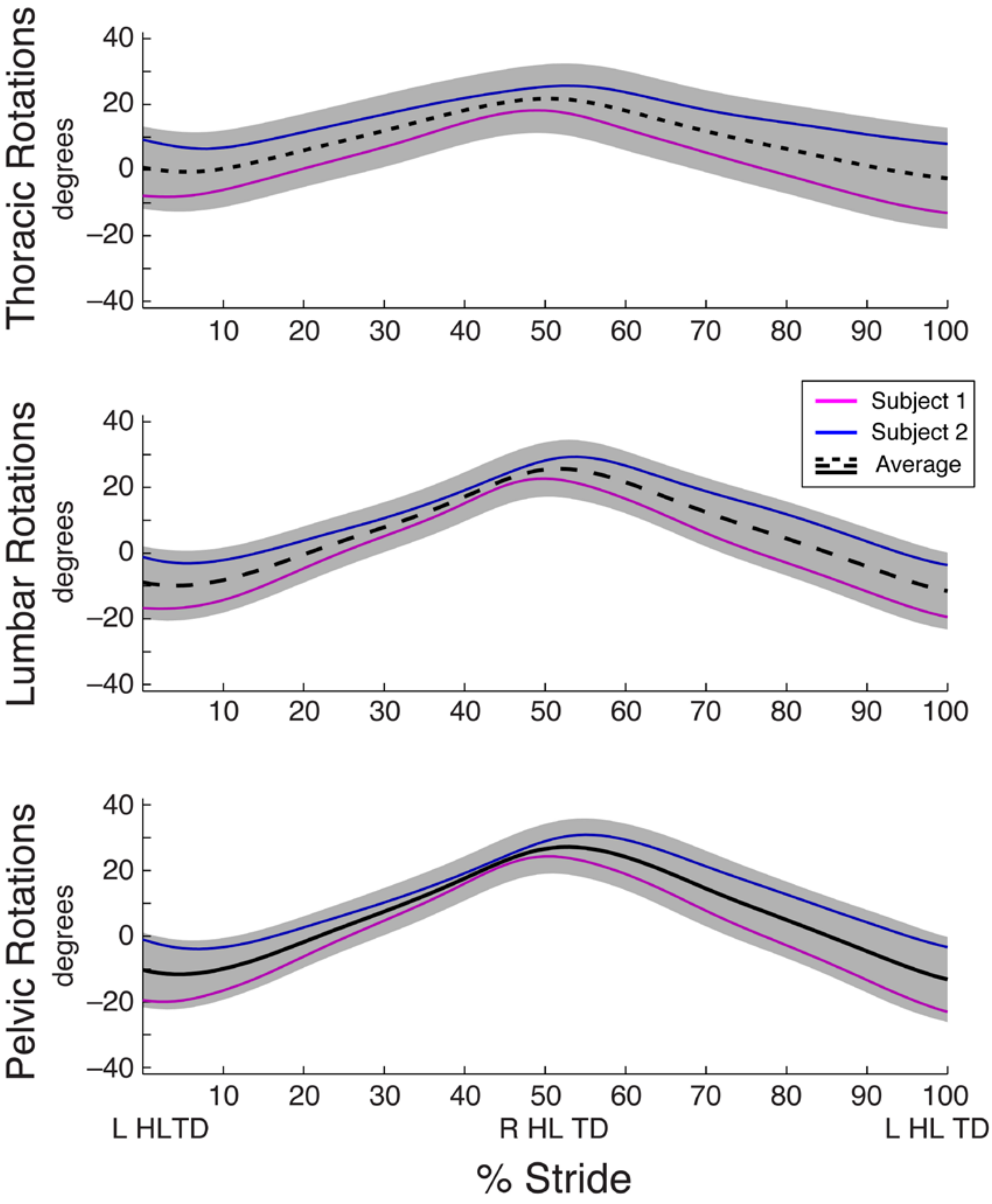

Supplementary Figure 2 | Mean angular motion of all segments for individual chimpanzee subjects. Rotations are relative to a global coordinate system. Colored lines represent individual chimpanzee subject means, and the thick black line represents the mean for all chimpanzee strides. Shaded area represents ± one standard deviation.

Supplementary Tables

Supplementary Table 1 | Kinematic variables for human subjects

	**Human Subject 1**		**Human Subject 2**		**Human Subject 3**		**Human Subject 4**		**Human Subject 5**	
	mean	st. dev.	mean	st. dev.	mean	st. dev.	mean	st. dev.	mean	st. dev.
Sex	Female		Female		Female		Female		Female	
Mass (*kg*)	53.2		60.2		52.5		48.1		65.0	
Age (*years*)	26.5		26.3		25.4		28.0		23.8	
Lower limb length (*m*)	0.83	*0.00*	0.88	0.00	0.88	0.01	0.82	0.00	0.84	0.01
Speed ($m\ s^{-1}$)	1.28	*0.04*	1.42	0.06	1.26	0.04	1.33	0.07	1.37	0.05
Dimensionless velocity	0.45	*0.02*	0.48	0.02	0.43	0.01	0.47	0.02	0.48	0.02
Number of strides	5		5		5		5		5	
Axial Range of Motion (°)										
Pelvis	11.9	*2.7*	13.3	4.0	12.2	1.9	11.9	2.3	18.6	1.6
Lumbar	11.2	*2.4*	11.6	3.6	13.4	1.3	10.4	2.1	12.3	2.5
Thorax	10.6	*2.2*	14.4	3.1	13.6	2.3	10.5	1.3	8.4	1.8
Thorax relative to pelvis	13.1	*1.8*	21.5	3.5	14.0	2.7	17.0	1.6	24.7	2.2
Lumbar relative to pelvis	5.2	*2.2*	5.2	0.5	4.6	0.5	6.6	0.7	6.6	0.9
Thorax relative to lumbar	12.0	*2.5*	18.0	2.8	11.3	3.1	10.5	0.7	17.5	2.2

	**Human Subject 6**		**Human Subject 7**		**Human Subject 8**		**Human Subject 9**		**Human Subject 10**	
	mean	st. dev.	mean	st. dev.	mean	st. dev.	mean	st. dev.	mean	st. dev.
Sex	Male		Male		Male		Male		Male	
Mass (*kg*)	66.8		61.5		66.0		68.7		73.1	
Age (*years*)	29.2		27.1		21.4		28.7		21.7	
Lower limb length (*m*)	0.93	0.00	0.87	0.00	0.90	0.00	0.88	0.00	0.96	0.00
Speed ($m\ s^{-1}$)	1.47	0.04	1.34	0.04	1.33	0.01	1.39	0.01	1.44	0.08
Dimensionless velocity	0.49	0.01	0.46	0.01	0.45	0.00	0.47	0.00	0.47	0.03
Number of strides	5		5		5		5		5	
Axial Range of Motion (°)										
Pelvis	9.9	0.6	13.6	2.4	10.9	1.7	8.3	1.7	16.4	2.2
Lumbar	7.4	1.3	9.0	2.4	7.4	2.0	8.2	1.2	10.5	2.2
Thorax	6.9	0.8	9.0	0.5	7.5	0.7	7.1	1.9	8.4	1.4
Thorax relative to pelvis	14.2	0.9	17.5	1.7	16.0	1.2	13.5	1.1	20.0	2.5
Lumbar relative to pelvis	5.0	1.3	7.2	0.5	6.5	0.9	7.4	0.7	6.8	0.8
Thorax relative to lumbar	10.8	1.8	12.3	2.0	11.3	1.2	9.3	1.5	14.0	2.4

	**Females**		**Males**		**P-value**
	mean	st. dev	mean	st. dev.	
Mass (*kg*)	55.8	6.1	67.2	3.9	**0.02**
Age (*years*)	26.0	1.4	25.6	3.5	0.84
Lower limb length (*m*)	0.85	0.02	0.91	0.03	**<0.001**
Speed ($m\ s^{-1}$)	1.33	0.08	1.39	0.07	**<0.01**
Dimensionless velocity	0.46	0.03	0.47	0.02	0.30
Number of strides	25		25		
Axial Range of Motion (°)					
Pelvis	13.6	3.6	11.8	3.4	0.07
Lumbar	11.8	2.5	8.5	2.1	**<0.001**
Thorax	11.5	3.0	7.8	1.4	**<0.001**
Thorax relative to pelvis	18.0	5.0	16.2	2.8	0.26
Lumbar relative to pelvis	5.7	1.3	6.6	1.2	**0.01**
Thorax relative to lumbar	13.8	3.9	11.5	2.3	0.05

P-values are results of Wilcoxon rank-sum tests with a sample size of 25 for each males and females.

Supplementary Table 2 | Kinematic variables for chimpanzee subjects

	**Chimpanzee Subject 1**		**Chimpanzee Subject 2**	
	mean	*st. dev.*	mean	*st. dev.*
Mass (*kg*)	35.7	*0.7*	34.2	*0.7*
Age (*years*)	7.0		7.1	
Lower limb length (*m*)	0.44	*0.01*	0.42	*0.01*
Speed ($m\ s^{-1}$)	0.94	*0.09*	1.00	*0.09*
Dimensionless velocity	0.45	*0.04*	0.49	*0.04*
Number of strides	7		7	
Axial Range of Motion (°)				
Pelvis	49.0	*10.8*	36.6	*5.5*
Lumbar	43.5	*11.9*	35.5	*5.1*
Thorax	32.5	*11.4*	21.2	*3.4*
Thorax relative to pelvis	19.9	*4.0*	18.2	*5.3*
Lumbar relative to pelvis	7.4	*2.8*	5.6	*4.8*
Thorax relative to lumbar	15.6	*3.0*	15.6	*2.4*

Mass includes standard deviations since each chimpanzee includes data from two recording sessions within a period of four months.

Supplementary Table 3 | Marker positions for trunk segments

	Humans	Chimpanzees
**Thorax markers**	First thoracic vertebra	
	Last thoracic vertebra	
	Anterolateral aspect of second (females) or fourth (males) rib	
**Lumbar markers**	First lumbar vertebra	
	Two markers on either side of third lumbar vertebra	
**Pelvic markers**	Left anterior superior iliac spine	
	Left and right posterior superior iliac spines	
	Left iliac blade	*Left ischial tuberosity*
		Sacrum
		Coccyx

The pelvic markers in *italics* represent those markers that were species specific.
